# Polyphenol extract of Phyllanthus emblica (PEEP) induces inhibition of cell proliferation and triggers apoptosis in cervical cancer cells

**DOI:** 10.1186/2047-783X-18-46

**Published:** 2013-11-19

**Authors:** Xinxian Zhu, Jianjun Wang, Yang Ou, Weiwei Han, Huaifang Li

**Affiliations:** 1 Department of Obstetrics and Gynecology, Tongji Hospital, Tongji University, No.389 Xincun Road, Shanghai 200065, China

**Keywords:** *Phyllanthus emblica* polyphenol extract, Cervical cancer, Cell cycle arrest, Apoptosis

## Abstract

**Background:**

The aim of this study is to investigate the effects of polyphenol extract from *Phyllanthus emblica* (PEEP) on cervical cancer cells and to explore the underlying mechanism.

**Methods:**

MTT assay was used to measure inhibition of proliferation of cervical cancer (HeLa) cells after treatment with PEEP at concentrations of 0, 50, 100, 150, and 200 mg/ml for 48 hours. HeLa cells were treated with PEEP (150 mg/ml) for 48 hours in the following analysis. Karyomorphism was assessed by immunofluorescence using DAPI staining, and cell apoptosis and cell cycle were assessed using flow cytometry. Three apoptotic marker proteins, namely, Fas, FasL, and cleaved caspase-8, were assessed by western blotting.

**Results:**

PEEP inhibited the growth of HeLa cells, and the optimum concentration of PEEP was 150 mg/ml. In addition, the karyomorphism of HeLa cells after treatment with PEEP was abnormal. Furthermore, PEEP induced arrest of the HeLa cell cycle at G2/M phase, and triggered apoptosis. PEEP also induced significant Fas and FasL activation, and cleavage of caspase-8.

**Conclusions:**

Our study indicates that PEEP is effective in inhibiting HeLa cell proliferation by inducing cell cycle arrest at G2/M phase and inducing apoptosis.

## Background

Cervical cancer is ranked as the second leading cause of female cancer mortality worldwide, with an annual incidence of approximately 200,000 deaths and more than 500,000 new cases diagnosed [1-3]. The incidence of cervical cancer is high in developing countries, and more than 28.8% of the world’s cases occur in China [4].

Human papillomavirus (HPV) infection is considered the greatest risk factor in the development of cervical cancer [5]. Although HPV vaccines have been licensed in several areas, such as the USA, Europe, Canada, and Australia, the incidence of HPV infection-related cervical cancer has not been eliminated [6]. This is because the vaccines are effective only against some types of HPV, and they are not yet widely used in developing countries [7]. Curative surgery is the first option for patients with early-stage cervical cancer, while radiotherapy and chemotherapy have proven to be effective treatments for patients in the advanced stages. However, the curative effect of traditional chemotherapeutic drugs is limitedm and their side effects, such as neurological and/or renal [8] and cardiac [9] toxicity, are serious. Therefore, research into novel chemotherapeutic drugs is essential for effective treatment of cervical cancer.

*Phyllanthus emblica* (PE; syn. *Emblica officinalis*, also known as the Indian or Nepalese gooseberry or emblic leaf-flower) is a species belonging to the family Euphorbiaceae, which is used as a traditional medicine, especially in Asia [10]. A previous study showed that PE could induce apoptosis in mouse and human carcinoma cell lines, including Dalton’s lymphoma ascites and CeHa cell lines [11]. In addition, Ngamkitidechakul *et al.* reported that PE was able to inhibit proliferation of a series of cancer cell lines, including A549, HepG2, HeLa, MDA-MB-231, SK-OV3, and SW620, suggesting potential for PE in oncotherapy [12].

The ingredients of PE are complex, and include tannin and phenolic glycosides, flavonoids, terpenes, sterols, and several human essential trace elements such as vitamins and amino acids [10]. In the present study, we isolated polyphenol extract from PE (PEEP), and measured its effect on the proliferation, cell cycle and apoptosis of cervical cancer (HeLa) cells. We also assessed karyomorphism of the cells after incubation with PEEP for 48 hours, and assessed expression of three apoptotic marker proteins: Fas, FasL, and cleaved caspase-8, using western blotting.

## Methods

### Preparation of PEEP

Polyphenols were extracted from the leaves of PE plants as described previously [13]. Briefly, the leaves were homogenized for 5 minutes with chilled 70% acetone, followed by homogenization at high speed for 5 minutes, then the homogenate was centrifuged for 10 minutes. This process was performed in triplicate. Finally, the extract was dissolved in dimethyl sulfoxide (DMSO, Sigma, St Louis, MO,USA) and stored at −20°C until used.

### Cell culture

HeLa cells were obtained from the Cell Bank of Type Culture Collection of the Chinese Academy of Sciences, (Shanghai, China), and were maintained in RPMI1640 (Gibco, Uxbridge, UK) with 10% fetal bovine serum (Hyclone, UT, USA) at 37°C in an atmosphere of 5% CO_2_. HeLa cells in the logarithmic phase were seeded into 96-well tissue culture plates at a density of 1 × 10^5^ cells per well, and allowed to grow for 24 hours before being treated with PEEP. Cells were exposed to different concentrations (50, 100, 150, and 200 mg/ml) of PEEP, with phosphate-buffered saline (PBS) used as a negative control.

### Proliferation assay

Cells were incubated for 48 hours at 37°C, then 20 μl MTT (3-(4, 5-dimethylthiazol-2-yl)-2, 5-diphenyltetrazolium bromide, 5 mg/ml; Sigma, St Louis, MO, USA) was added to each well, and cells were maintained at 37°C for a further 4 hours. After this, 150 μl DMSO was added to each well, and the optical density (OD) of each well at 570 nm was measured by a microplate reader (Thermo Fisher Scientific, Waltham, MA). All experiments performed in triplicate. Inhibition rate was calculated by the following formula:

$$\begin{array}{l} \text{inhibition}\;\text{rate}=[1-\text{OD}(\text{PEEP})÷\text{OD}(\text{PBS})]\\ \;\times 100\%. \end{array}$$

### Immunofluorescence assay

HeLa cells in the logarithmic phase that had been treated with PEEP (150 mg/ml) for 48 hours were seeded at a density of 2 × 10^5^ cells onto coverslips, and maintained at 37°C in an atmosphere of 5% CO_2_ for another 48 hours. After that, cells were washed three times with PBS, and fixed in 3.7% paraformaldehyde for 20 minutes. The nuclei were stained with DAPI (4′, 6-diamidino-2-phenylindole; Roche, Mannheim, Germany) for 15 minutes. Images were captured using a confocal microscope.

### Cell cycle analysis

HeLa cells in the logarithmic phase were seeded on a 96-well plate at a density of 1 × 10^5^ cells per well and treated with PEEP (150 mg/ml), with PBS used as a control. At 48 hours after treatment, the cells were harvested and washed twice with PBS. The cells were then stained with 0.1% Triton X-100 containing propidium iodide (Amersham, Buckinghamshire, UK) and RNase (NEB, Ipswich, USA). Fluorescence from the propidium iodide-DNA complex was measured by flow cytometry (Millipore, Billerica, USA).

### Analysis of apoptosis

For analysis of cell apoptosis, HeLa cells in the logarithmic phase were seeded at a density of 1 × 10^5^ cells per well and treated with PEEP (150 mg/ml) for 48 hours before harvesting. Cells were washed twice with PBS and resuspended in PBS at a density of 1 × 10^5^ cells/ml. Before being analyzed by fluorescence-activated cell sorting (FACS) (FACSCaliburl Milipore, Billerica, USA), 100 μl of cells were mixed with 100 μl Guava Nexin reagent (Milipore, Billerica, USA) for 20 minutes. All experiments were carried out in triplicate.

### Western blotting

Cells in the logarithmic phase were harvested and lysed on ice for 30 minutes. After centrifugation at 2,000 rpm (626 g) for 10 minutes, the supernatant was collected, and protein concentrations were determined by bicinchoninic acid assay (BCA). Proteins were separated by 10% SDS-PAGE (20 μg protein per well). Then proteins were transferred to PVDF membrane (Bio-Rad, Hercules, USA). The membrane was blocked in Tris-buffered saline containing 5% non-fat milk (Wyeth) for 2 hours, and subsequently incubated with primary caspase-8 (1:200 dilution), Fas (1:200 dilution), FasL(1:200 dilution), and GAPDH antibodies (Abcam, Cambridge, USA) overnight at 4°C, followed by incubation with secondary antibody (Abcam, Cambridge, USA) at room temperature for 1 hour. The proteins of interest were visualized using an enhanced chemiluminescence (ECL) system (Millipore). GAPDH expression was used as internal control.

## Results

### PEEP inhibits HeLa cell proliferation

**Figure 1:**
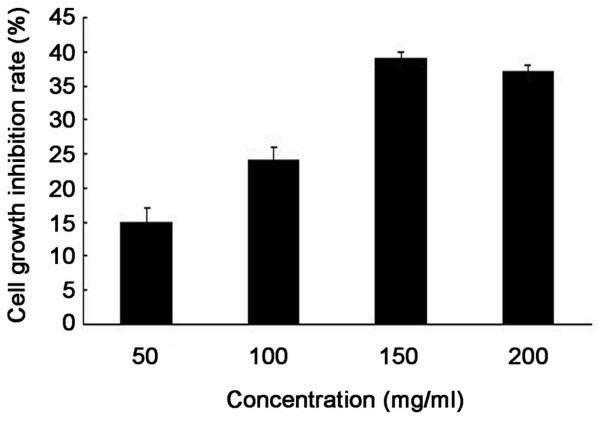
**Polyphenol extract of *Phyllanthus emblica *(PEEP) inhibits HeLa cell proliferation.** HeLa cells were treated with different concentrations of PEEP (50, 100, 150, 200 mg/ml) for 48 hours. Quantification of the number of viable cells was determined by MTT assay. The data are expressed as the means ± SD obtained from three independent experiments.

To investigate the effect of PEEP on HeLa cell proliferation, we assessed the inhibition rate of HeLa cells after PEEP treatment. PEEP inhibited proliferation in a dose-dependent fasion up to 200 mg/ml, after which it decreased. The maximum inhibition rate was 39% when the PEEP concentration was 150 mg/ml (Figure 1).

### PEEP induces G2/M arrest of HeLa cells

**Figure 2:**
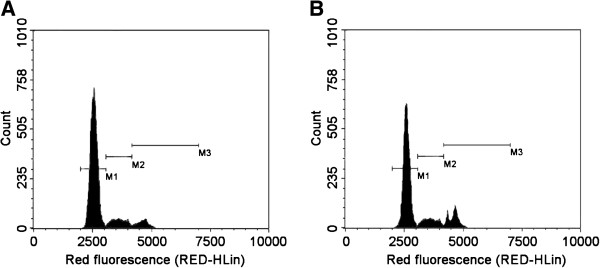
**Polyphenol extract of *Phyllanthus emblica *(PEEP) induces cell cycle arrest of HeLa cells at G2/M phase.** HeLa cells were treated with 0 mg/ml PEEP **(A)**, 150 mg/ml PEEP **(B)** for 48 hours, and the cell cycle stage was determined by flow cytometry. The panels of M1, M2, and M3inside the image are the marks of stage borders of G_0_/G_1_, S, and G_2_/M, respectively.

To explore the role of PEEP in cell cycle regulation, we treated HeLa cells with 150 mg/ml PEEP for 48 hours, and found that cell cycle arrest was induced at the G2/M phase after PEEP treatment. The number of HeLa cells in G2/M was 10% with PBS, and this increased to 31% after PEEP treatment. However, the number of cells in G1 phase decreased from 65% to 25%, and those in the S phase decreased from 51% to 18% (Figure 2). These results suggest that PEEP is able to induce arrest of HeLa cells in G2/M phase.

### PEEP changes the karyomorphism of HeLa cells

**Figure 3:**
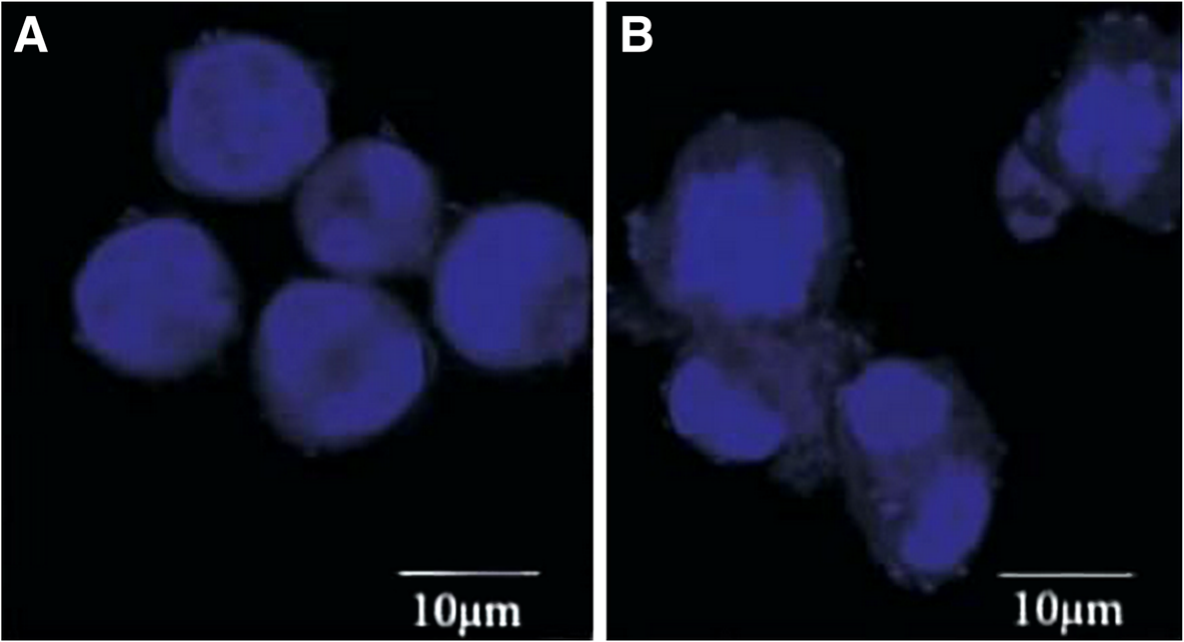
**Polyphenol extract of *Phyllanthus emblica *(PEEP) changes the karyomorphism of HeLa cells.** HeLa cells were seeded onto coverslips, and treated with 0 mg/ml PEEP **(A)**, 150 mg/ml PEEP **(B)** for 48 hours before harvesting. DAPI staining was used to highlight the nucleus. The panels inside the image represent the size of 10 μm.

The significant effects of PEEP on HeLa cell proliferation and cell cycle arrest led us to consider its effect on the karyomorphism of HeLa cells. Therefore, we performed immunofluorescence experiments of HeLa cells treated with PEEP. In normal cells, the chromatin was well distributed and the karyotheca were integrated. By contrast, after treatment with PEEP for 48 hours, the chromatin was condensed and the karyotheca were ruptured (Figure 3).

### PEEP induce apoptosis of HeLa cells

**Figure 4:**
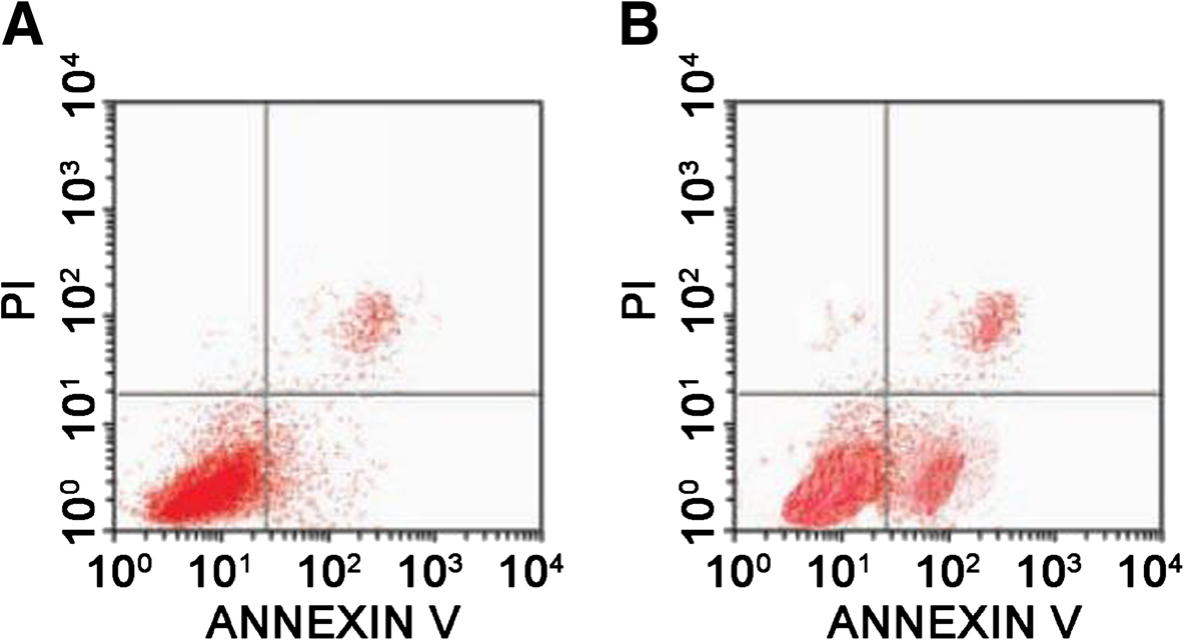
**Polyphenol extract of *Phyllanthus emblica *(PEEP) induces HeLa cell apoptosis.** HeLa cells were treated with 0 mg/ml PEEP **(A)**, 150 mg/ml PEEP **(B)** for 48 hours. The cells were harvested and resuspended in Guava Nexin. Cell cycle stage was determined by flow cytometry. The panels inside the image are marks of the quadrants, cells in the left lower quadrant are living cells, in the right upper quadrant are dead cells, and in the right lower quadrant are apoptotic cells.

To identify the functional role of PEEP in mediating apoptosis of HeLa cells, the apoptosis rate of HeLa cells after PEEP treatment was determined by FACS. The rate of apoptosis increased from 4.8 ± 2.1% to 45 ± 2.4% after PEEP treatment (Figure 4).

**Figure 5:**
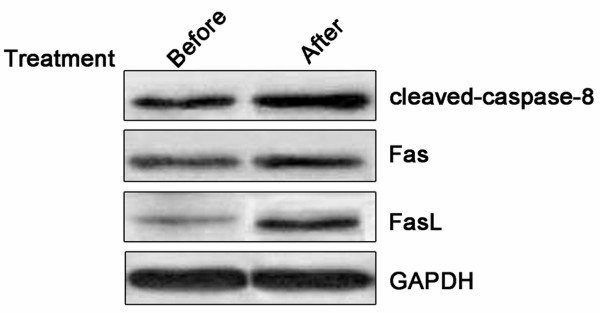
**Polyphenol extract of *Phyllanthus emblica *(PEEP) induces activation of Fas, FasL, and cleaved caspase-8.** Western blotting analysis of Fas, FasL, and cleaved caspase-8 (Δ-caspase-8), in HeLa cells exposed to PEEP for 48 hours.

In order to understand the mechanism underlying this effect of PEEP on apoptosis of HeLa cells, expression of three apoptotic marker proteins (Fas, Fas L and clevaved caspase-8 were analyzed by western blotting. PEEP treatment induced significant Fas and FasL activation, and cleavage of caspase-8 (Figure 5). Based on these results, we concluded that PEEP is able to activate the cell apoptosis pathway in HeLa cells.

## Discussion

PE is a traditional medicine that has been investgated fo antiviral [14,15] and anti-cancer [12] propertied, with satisfactory results. Although PE seems to have an anti-cancer effect, the active ingredients of PE are not very clear. The structure of PEEP contains an aromatic nucleus, one or more phenolic hydroxyl groups, and other elements. In the present study, we isolated polyphenols from PE and determined that in HeLa cells, this extract is capable of inhibiting cell proliferation, inducing cell cycle arrest at G2/M phase, and triggering apoptosis. We suggest that these effects were mainly due to the unique characteristics of polyphenols.

There are two common mechanisms identified in drug-induced inhibition of cancer cell proliferation [16-18]. First, chemotherapeutic drugs induce cell cycle arrest, especially in S or G2/M phase, with consequent inhibition of cell growth. Second, such drugs trigger the apoptotic pathway in cells. Fas, FasL, and caspase-8 are all molecular markers in the apoptotic pathway [19]. Zhang *et al*. indicates that polyphenols extracted from fruit juice or from PE leaves had a strong inhibitory effect on melanoma cells [20]. In addition, PEEP is efficient in facilitating the cytotoxicity of other drugs, such as doxorubicin and *cis*-platinum, when co-administered [21]. In the current study, we determined that the inhibition of HeLa cell proliferation increased as PEEP concentration increased, reaching a maximum inhibition level of 39% at 150 mg/ml PEEP. Further experiments showed that PEEP inhibited HeLa proliferation by inducing cell cycle arrest at G2/M phase and triggering apoptosis.

It has been reported previously that PEEP induced G2/M phase arrest of lung cancer cells and triggered cell apoptosis [22]. Our data suggest that PEEP similarly induces HeLa cell cycle arrest at G2/M phase. Further experiment showed that cell apoptosis was also triggered. Consistent with this, three apoptotic markers, Fas, FasL and cleaved caspase-8, were increased, suggesting that PEEP inhibits cell proliferation by inducing cell cycle arrest at G2/M phase and triggering apoptosis in cervical cancer cells.

## Conclusions

PEEP was able to inhibit proliferation and promote apoptosis of cervical cancer HeLa cells by inducing cell cycle arrest at G2/M phase and triggering apoptosis. PEEP may be a potential future chemotherapy drug with definite functional components.

## Competing interests

The authors declare that they have no competing interests.

## Authors’ contributions

XXZ and HFL designed the experiments: XXZ, YO, and WWH performed and interpreted the experiments: and JJW wrote the manuscript. All authors read and approved the final manuscript.
